# Investigation on the Interaction between Cellulosic Paper and Organic Acids Based on Molecular Dynamics

**DOI:** 10.3390/molecules25173938

**Published:** 2020-08-28

**Authors:** Mengzhao Zhu, Chao Gu, Wenbing Zhu

**Affiliations:** Equipment Status Evaluation Center, State Grid Shandong Electric Power Research Institute, Jinan 250002, China; guch2000@163.com (C.G.); zhwebing@163.com (W.Z.)

**Keywords:** organic acids, crystalline cellulose, molecular dynamics, adsorption

## Abstract

Organic acid is an important factor that accelerates the aging of cellulosic insulation materials. In this study, the interactions between cellulose and five acids, representative of what may be found in an aging transformer, were studied using molecular dynamics. The adsorption process of the five acids onto the surface of crystalline cellulose shows that the three low molecular acids are more readily adsorbed onto cellulose than the two high molecular acids. The deformation and adsorption energies of the acids all increase with an increase in molecular weight when they are stably interacting with cellulose. However, the differences between adsorption energies and deformation energies are positive for the three low molecular acids, whereas they are negative for the two high molecular acids. This indicates that the attachments onto cellulose of low molecular acids are considerably more stabilized than those of the high molecular acids. This is consistent with the experimental results. Furthermore, based on the calculated solubility parameters of acids, the experimental result that the three low molecular acids are to a large degree absorbed onto the cellulose, whereas the two high molecular acids remain in the oil, was theoretically elucidated using the theory of similarity and intermiscibility.

## 1. Introduction

Cellulosic paper is a major component of the transformer insulation system. It deteriorates with age, leaving the insulation vulnerable to stresses generated by thermal, mechanical and electrical transients [1,2,3]. Water, heat and oxygen all contribute to the degradation process. The aging of cellulose can be due to three different processes: pyrolysis, oxidation, and hydrolysis. Acid-hydrolysis is essentially one of the depolymerisation processes, under a synergism between water and hydrogen ions [4,5], and the rate of hydrolysis is proportional to the amount of hydrogen ions.

Lundgaard et al. found that carboxylic acids are aging products of oil-paper insulation materials. Low molecular acids (formic acid, acetic acid and levulinic acid) are formed by aging of cellulose, and high molecular acids (naphtenic acid and stearic acid) are formed by aging of insulating oils. Low molecular acids and water have a clear synergy in accelerating the aging of insulating paper, whereas high molecular acids do not influence paper aging significantly. Low molecular acids are readily absorbed by cellulose paper, contrary to high molecular acids that remain in oil [6]. N. Azis et al. conducted aging experiments and found that low molecular acids significantly accelerated the aging rate of insulating paper, while the aging rate of insulating paper with added high molecule acids and without added acid samples was basically the same [7]. Ingebrigsten et al. found that formic acid, acetic acid and levulinic acid, formed by the degradation of cellulose, are absorbed by paper, and the content of low molecular acids in cellulose insulation paper is about 100 times that of low molecular acids in oil. The acidity mainly comes from the high molecule acids, while the low molecule acids in oil only account for 10–15% [8]. The low molecular acids in cellulose paper are present in dozens or even hundreds times the amount of those in oil [9,10].

Although the effects of different organic acids on the aging of cellulose have been widely studied, most relevant studies have been confined to laboratory experiments due to the complexity of the physiochemical process of the aging of cellulose. Investigations on the micro-mechanism underlying the influence of acids on the aging of cellulose remain at the exploratory stage. Therefore, the study of the different effects of different acids on the aging of cellulosic paper at the microscopic level can help shed light on the aging mechanism of the oil-paper insulation of transformers.

In recent years, with the improvements in computer technology and computational methods, molecular simulation has developed into an effective means of analyzing complex systems at the molecular level [11,12,13]. Molecular simulation can reveal detailed information regarding organic molecular structures, and is a powerful tool to complement experiments. It helps in examining the micro-mechanisms of the aging process of cellulosic paper caused by organic acids.

## 2. Results and Discussion

### 2.1. Adsorption of Acid Molecules

**Figure 1:**
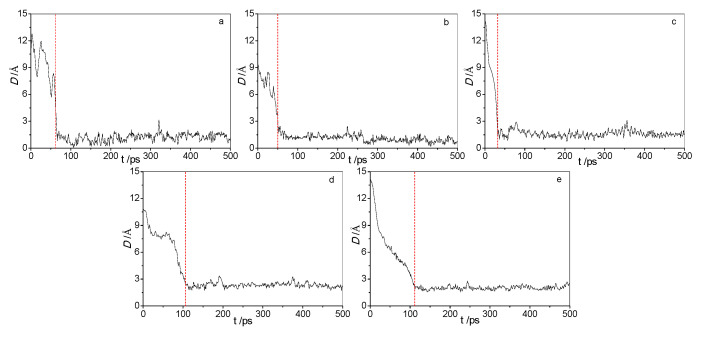
The distances of the centroids of five acid molecules from the surface as a function of simulation time. (**a**) Formic acid; (**b**) Acetic acid; (**c**) Levulinic acid; (**d**) Naphtenic acid; (**e**) Stearic acid.

**Figure 2:**
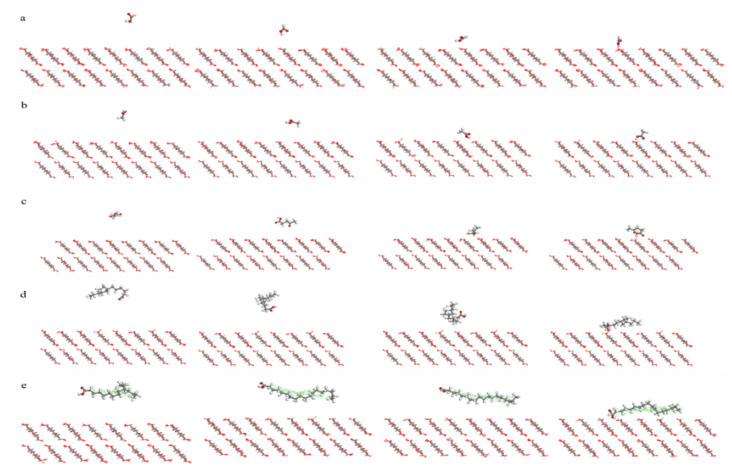
Adsorption process of different acid molecules at 0 ps, 25 ps, 50 ps and 100 ps. (**a**) Formic acid; (**b**) Acetic acid; (**c**) Levulinic acid; (**d**) Naphtenic acid; (**e**) Stearic acid.

Figure 1 shows the adsorption process of the five acid molecules. As shown in the Figure 1a–e, all five acid molecules were quickly adsorbed onto the crystal with an increase in simulation time. However, the instances where the three low molecular acids were adsorbed completely onto the surfaces and stably remained on the surface went through about 50 ps, whereas the two high molecular acids went through nearly 100 ps. This shows that the three low molecular acids can easily be adsorbed onto the cellulose surface because of their smaller momentum, whereas the two high molecular acids had great difficulty in reaching the surface. Considering the oil environment in a transformer, the two high molecular acids exerted a stronger steric effect than the three low molecular acids. This made it more difficult for the two high molecular acids to reach the cellulose surface. Figure 2 shows the dynamic diagram of the adsorption process of five acid molecules at different times. It can be seen from the Figure 1 that all three low molecular acids were adsorbed by cellulose at 50 ps, while the two high molecular acids were not well adsorbed by cellulose.

**Figure 3:**
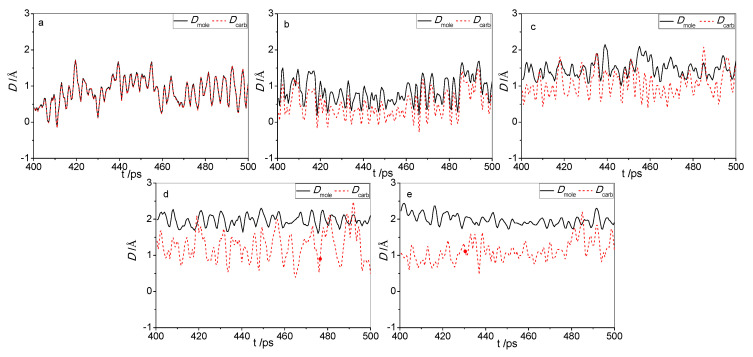
The distance of the five acid molecules (*D_mole_*) and their carboxyl groups (*D_carb_*) from the surface as a function of simulation time. (**a**) Formic acid; (**b**) Acetic acid; (**c**) Levulinic acid; (**d**) Naphtenic acid; (**e**) Stearic acid.

There is one carboxyl group in each of the five acids, which is a typical polar group. Because cellulose is polar as well, the adsorption potential of the carboxylic group with cellulose is definitely higher than those of other non-polar groups with cellulose. Moreover, the active group for all the five organic acids is the carboxyl group; hence, the sites where the carboxyl groups were adsorbed are very significant. Figure 3 shows the respective distances of the centroids of the five acid molecules (*D_mole_*) and the centroids of the carboxyl groups (*D_carb_*) from the surface change with the simulation time in the final 100 ps of the MD. As shown by the graphs, the splits between the curves of the two centroids become increasingly obvious with an increase in molecular weight of the acid.

Table 1 shows the respective distances of the two centroids from the surface when the acid molecules were interacting stably with cellulose. If the carboxyl groups and other parts of the acid molecules have the same adsorption interaction with cellulose, the two centroids should coincide. However, as shown in the table, the distances of the two centroids from the surface are not equal to each other. Both the carboxyl group and cellulose are polar, whereas other parts (namely the alkyl chain except for levulinic acid) are non-polar. Therefore, the interaction between the carboxyl group and cellulose is much stronger than that between other parts and cellulose, making the carboxyl group much closer to the cellulose surface than other parts. Meanwhile, the data in the table clearly show that *D_mole_* increased with an increase in its molecular weight, as well as the difference between the distances of the two centroids (*D_diff_* = *D_mole_* − *D_carb_*). This indicates that the carboxyl group is the stable adsorbed point, whereas other parts of the acid molecule were adsorbed unstably. The greater the molecular weight of the organic acid, the looser its adsorption onto cellulose.

**Figure 4:**
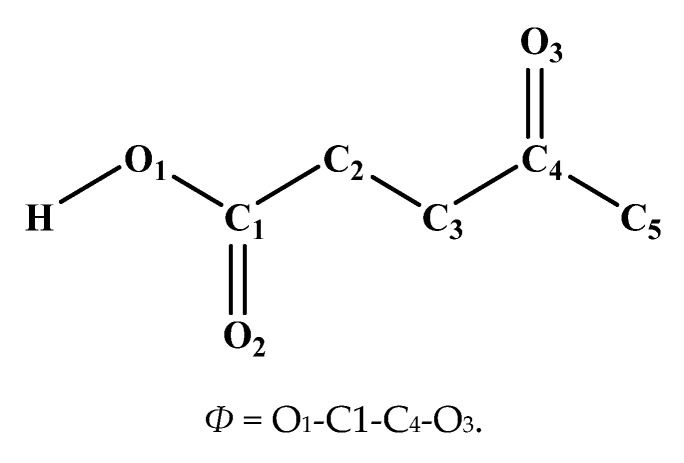
The dihedral angel formed between the carboxyl group and carbonyl group of levulinic acid.

Levulinic acid has an additional carbonyl group aside from the carboxyl group compared with the four other organic acids, as shown in Figure 4. The carbonyl oxygen atom can form hydrogen bonds with cellulose, which would increase adhesion to the cellulose surface; however, its carboxyl group can also form hydrogen bonds with cellulose, causing spatial competition between the carboxyl group and the carbonyl group. Figure 4 shows that the distances of the centroids of both the levulinic acid molecule and the carbonyl oxygen atom from the surface vary with the simulation time in the last 100 ps of MD. The changes in the dihedral angle defined in Figure 4 with the simulation time are given simultaneously.

**Figure 5:**
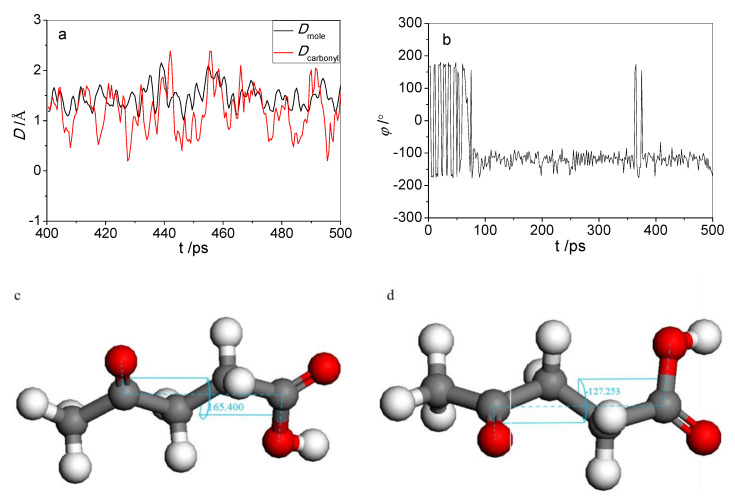
(**a**) Distance of the carbonyl group and the molecular centroid from the cellulose surface varies over time; (**b**) Dihedral angle between the carbonyl group and the carboxyl group varies over time; (**c**) Dihedral angles of carbonyl and carboxyl groups before adsorption; (**d**) Dihedral angles of carbonyl and carboxyl groups after adsorption.

As shown in Figure 5a, the distance of the carbonyl oxygen atom from the surface (*D_carbonyl_*) is smaller than that between the centroid of the levulinic acid molecule from the surface (*D_mole_*) most of the time. From Figure 5b, before the levulinic acid molecule was adsorbed onto the surface of cellulose, the dihedral angle was about 180°, indicating that the carbonyl group and the carboxyl group lay in opposite directions. When the levulinic acid molecule was stably absorbed onto the surface, the dihedral angle decreased to about 120°. This is mainly because the levulinic acid molecule underwent self-adjustment of the spatial structure under the influence of cellulose, and consequently, both the carbonyl and carboxyl groups got closer to the surface of cellulose and formed stronger interactions with it. However, the adjustment of the spatial structure will inevitably increase the potential energy. Whether the newly generated spatial structure can exist stably depends mainly on the competition between the deformation energy of the levulinic acid molecule and the adsorption energy of cellulose. Figure 5c shows the molecular structure of the levulinic acid molecule before it was adsorbed on the cellulose surface, the dihedral angle before the adsorption is marked. Analogously, Figure 5d shows the molecular structure of the levulinic acid molecule after it has been adsorbed on the cellulose surface, the dihedral angle after the adsorption is also marked.

### 2.2. Morphologies of the Adsorbed Acid Molecules

#### 2.2.1. Deformation Energy

The deformation energy represents the change in the bound and free state energy of an atom, ion, or molecule during the deformation process. Apart from the levulinic acid molecule, the other four organic acid molecules were deformed when they were adsorbed onto the surface of cellulose. The degree of deformation can be characterized by the deformation energy as follows:

(1) $$E_{\mathrm{def}}=E_{\mathrm{bind}}-E_{\mathrm{free}}$$

where *E*_bind_ and *E*_free_ are the energies of the bound state and free state of the acid molecule, respectively.

As shown in Table 2, due to the adsorption of cellulose, all five acid molecules have varying different degrees of deformation. The deformation energy of the acid molecule increases with an increase in molecular weight. Moreover, the deformation energies of the three low molecular acids are quite different from those of the two high molecular acids. The deformation energy affects the adsorption stability. In general, the higher the deformation energy exhibited, the more unstably the acid molecule is adsorbed.

#### 2.2.2. Adsorption Energy

The adsorption energy represents the decreasing energy produced by the bonding of two materials during the adsorption process of atoms, ions, or molecules attached to a substance surface. This energy is mainly used to calculate the chemical engineering properties and explore the adsorption mechanism. The adsorption energy of the acid molecule by cellulose can be calculated using the following formula:

(2) $$E_{\mathrm{ads}}=E_{1}+E_{2}-E_{12}$$

where *E*_12_ is the total energy of the system, *E*_1_ is the energy of acid molecules, and *E*_2_ is the energy of cellulose.

Table 3 gives the adsorption energies of the five organic acids when they were interacting stably with cellulose. As shown in the Table 3, the adsorption energy increases with an increase in the molecular weight of the acid. Moreover, the ratio of the van der Waals term to the coulomb term also varies with the change in the molecular weight of acid. As the carboxyl group and cellulose are Polar, the coulomb term dominates their adsorption energy. Hence, for an acid molecule, the higher the proportion that the carboxyl group accounts for, the higher the ratio of the coulomb term to the Van der Waals term is when it interacts with cellulose. For formic acid, the proportion of the carboxyl group is much higher, and the rate of the coulomb term is correspondingly greater. Based on the data shown in Table 1, the proportion of the carboxyl group decreases with an increase in the molecular weight of the acids. As a result, the coulomb term decreases, whereas the van der Waals term increases and becomes the most significant component of the adsorption energy. The increase in molecular weight of the acid molecule inevitably leads to an increase in adsorption energy, but this cannot possibly indicate that the acid molecule would be adsorbed onto the cellulose more steadily. As shown in Table 3, the adsorption energies decrease with the decrease of the acid molecular weight, and the low molecular weight acids are more inclined to be adsorbed by cellulose. This prediction is consistent with the experimental conclusions of Kouassi et al. [9].

Table 4 gives the adsorption energy densities of the five organic acids, D = *E*_ads_/V. The calculated and the experimental volumes [14] of the acid molecules are also given in this table. The calculated and the experimental volumes are approximate for the low molecular acid molecules but show differences for the high molecular acid molecules. Considering the matter of consistency, the calculated volume was chosen for the calculation of energy density for each of the five acids. The data show that the adsorption energy densities of the three low molecular acids are an order of magnitude higher than those of the two high molecular acids, although the adsorption energies of the latter are relatively larger. However, to determine whether the acid molecules can be adsorbed stably onto the surface of cellulose, the relation between the adsorption energy and the deformation energy need to be numerically evaluated. In addition, differences between the adsorption energy and the deformation energy can be shown in Table 4, where Δ*E* = *E*_ads_ − *E*_def_, for the five organic acids. As shown in Table 4, the differences of the three low molecular acids are positive, whereas for the two high molecular acids, the differences are negative. This indicates that the three low molecular acid molecules can be stably adsorbed onto the cellulose, whereas the two high-molecular acid molecules cannot. These results agree well with the experiments [4,6].

### 2.3. Solubility Parameter

The solubility parameter, proposed by Hildebrand et al. [15] in the mid-20th century, is defined as the square root of the cohesive energy density:

(3) $$\delta =\sqrt{\frac{CED}{V}}=\sqrt{\delta_{E}^{2}+\delta_{V}^{2}}$$

where *CED* represents cohesive energy density, which refers to the energy needed by 1 mol condensate per unit volume to overcome the gasification of intermolecular forces, and *V* represents molar volume.

The solubility parameter has been widely applied in polymer engineering and related fields as an important parameter for measuring the compatibility of the insulation materials.

According to the theory of similarity and intermiscibility, a solute can only be dissolved or swollen in a solvent that is of similar solubility to the solute. Table 5 shows the calculated and experimental solubility parameters of the five organic acids. The calculating details are as follows. Firstly, five amorphous cells were constructed. Each cell contained 200 molecules of the same acid. Other parameter settings, such as the force field, are the same as those mentioned above. Then, a 200 ps NPT dynamic run in which the temperature is maintained at 298 K was used. Other parameter settings are the same as the above molecular dynamic settings, namely, temperature was maintained using the Andersen algorithm. For the non-bonded interactions, the atom-based method [16,17] and the Ewald summation method [18,19] were employed to evaluate the electrostatic and van der Waals interactions, respectively. This is followed by an energy minimization.

As shown in the table, the calculated solubility parameters deviate from their experimental values in the low molecular acids, whereas they agree roughly with the experimental values in the high molecular acids. The solubility parameters of the three low molecular acids are approximate to the solubility parameter of cellulose in contrast to those of the two high molecular acids, which are more similar to that of oil. The result explains the experimental phenomenon that the low molecular acids are to a large degree absorbed by cellulose, contrary to the high molecular acids that tend to dissolve in the oil. For the Coulomb component of the solubility parameter, either its absolute value or proportion decreases with an increase in the molecular weight. Contrarily for the van der Waals component, although its absolute value decreases with an increase in the molecular weight, its occupied proportion indeed increases. The proportion of the polar group in the acid molecule again determines this change.

## 3. Calculation Methods

### 3.1. Modeling

**Figure 6:**
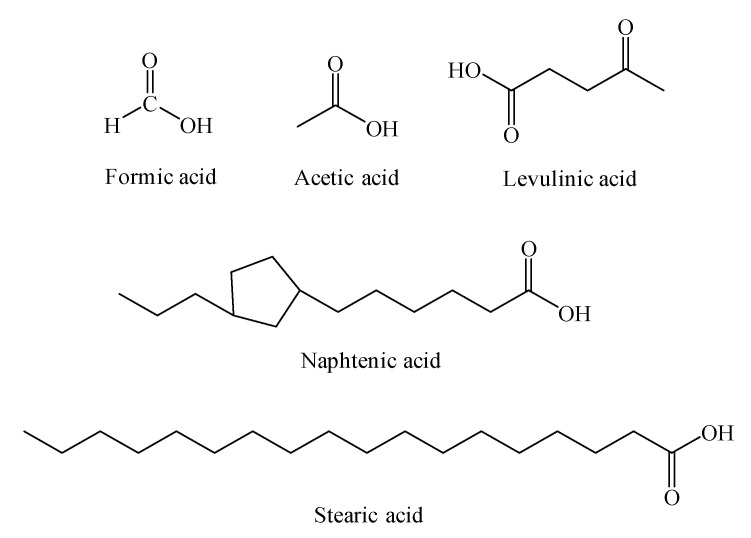
Diagrammatic sketches of acid molecules.

The five organic acids generated by the aging of oil-paper composites are shown in Table 6 and Figure 6. These models are constructed according to the sketch maps.

Cellulose, a linear polymer of glucose units linked to one another in a special manner, is the essential component of cellulosic paper [20,21]. There are two morphologies for cellulose in cellulosic paper, namely, crystalline and amorphous. The crystallinity of cellulose differs due to the sources of the cellulose, but it generally ranges from 60% to 80%, indicating that the surface of the crystal comprises a large portion of the whole surface of cellulose. The main raw material of cellulosic insulation paper is wood, which mostly contains cellulose Iβ crystal. Hence, in this study, the Iβ crystal presented by Nishiyama et al. [22] is adopted as the cellulose model.

**Figure 7:**
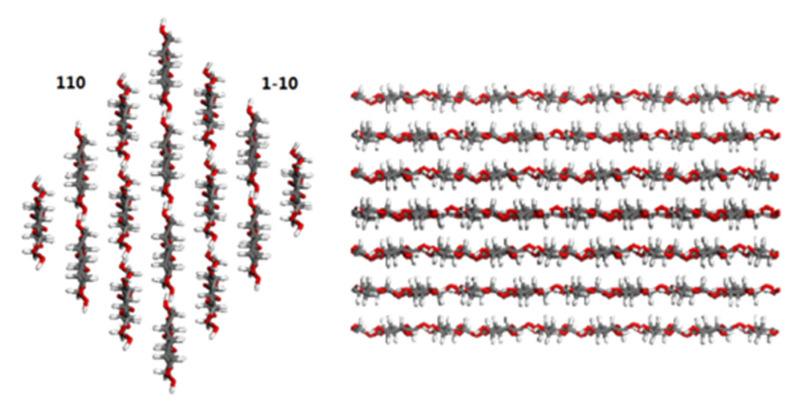
The crystalline cellulose model.

Many studies have found that different surfaces of the Iβ crystal exhibit different properties [23,24]. Atomic force microscopy [25,26,27,28] studies have revealed that the (110) and (1-10) surfaces make up a large part of cellulose nanocrystal surfaces. Both surfaces have numerous hydroxide radicals and similar adsorption behavior with organic matter. Therefore, the (1-10) surface was adopted as the adsorption surface. The super-cell was constructed following the construction of the (1-10) surface, which consists of 16 chains with eight residues each (Figure 7). The number of chains is fewer than the 36 chains expected for native cellulose samples in order to match the computational power available. However, the model is quite suitable because it exhibits the major ultrastructural characteristics of cellulose. The carbon-carbon and carbon-oxygen valences go across the cell boundary in the chain direction to mimic the infinite length of cellulose chains when periodic boundary conditions are used.

### 3.2. Simulated Details

Structural optimization was performed for the five organic acids, including annealing and energy optimization, to obtain their minimum energy conformations. Five models, with the same (1-10) surface and different acid molecules, were constructed. Only one acid molecule was added into the cell and placed at approximately 15 Å in front of the crystal surface for each model. Then it was let free to evolve under the lone influence of the cellulose. All cellulose chains were rigidly constrained to their initial positions except for the chains in the top layer. In fact, releasing all constraints would require a larger model that would entail more time for the simulation. Structural optimization and molecular dynamics (MD) were carried out using the second-generation polymer consistent force field (PCFF) [29,30,31,32,33] especially suited for carbohydrates and polymers [34,35]. The target temperatures of all MD were set to the practical transformer service temperature of 343 K. All simulations were carried out for 500 ps in the NVT ensemble. The standard Verlet algorithm was used to integrate Newton’s law of motion with a time step of 1 fs. Each molecular dynamics run was started by assigning initial velocity for the atoms according to a Boltzmann distribution. Temperature was maintained using the Andersen algorithm. For the non-bonded interactions, the atom-based method [16,17] and the Ewald summation method [18,19] were employed to evaluate the electrostatic and van der Waals interactions, respectively. All simulations were performed using Materials Studio 4.0 software developed by Accelrys Software Inc. (San Diego, CA, USA, http://accelrys.com/products/materials-studio/index.html).

## 4. Conclusions

The adsorption behaviors of the five organic acids in transformer oil-paper insulation materials were studied using molecular dynamics. The main conclusions are as follows.

The changes in distances of centroids of the five organic acid molecules from the surface of cellulose indicate that the three low molecular acids are more readily adsorbed onto cellulose than the two high molecular acids. The conformations of the acid molecules are greatly related to their polarities when adsorbed onto cellulose. The adsorption of acid molecules onto cellulose becomes increasingly unstable with an increase in the molecular weight of the acid molecules although all their carboxyl groups are stably adsorbed onto the cellulose.

All five acid molecules deformed when they were adsorbed onto the cellulose, with an increase in the molecular weight; both the deformation energy of the acid molecule and the absorption energy of cellulose increased. However, the adsorption energy density of each acid molecule decreased. The difference in adsorption energy and deformation energy shows that the three low molecular acids can be stably adsorbed onto cellulose, whereas the two high molecular acids cannot. This is consistent with the experimental results.

The solubility parameters of the three low molecular acids are approximate to that of cellulose, whereas the solubility parameters of the two high molecular acids are close to that of transformer oil. The result to some extent contributes to theoretically explain the experimental phenomenon that the low molecular acids are to a large degree absorbed by cellulose, contrary to the high molecular acids that tend to remain in the oil using the theory of similarity and intermiscibility.

## Figures and Tables

**Table 1:** The distances of centroids of acid molecules and carboxyl groups form the surface (Å).

	Formic	Acetic	Levulinic	Naphtenic	Stearic
*D_mole_*	15.16	15.16	15.77	16.27	16.28
*D_carb_*	15.14	14.79	15.36	15.56	15.42
*D_diff_*	0.01	0.37	0.41	0.71	0.86

**Table 2:** The deformation energies of acid molecules (kcal/mol).

	Formic	Acetic	Levulinic	Naphtenic	Stearic
*E*_free_	−33.7215	−56.9919	−49.7096	−66.4094	−94.1720
*E*_bind_	−29.2497	−51.8050	−35.5326	−20.9013	−15.4935
*E*_def_	4.4718	5.18689	14.1770	45.5081	78.6786

**Table 3:** Adsorption energies of acids when interacting stably with cellulose (kcal/mol).

	Formic	Acetic	Levulinic	Naphtenic	Stearic
*E*_ads_	11.4667	13.9769	19.4414	23.4770	26.7302
*E*_VdW_	4.8227	5.9117	9.9447	17.6817	23.3993
*E*_elec_	8.0650	6.6441	9.4967	5.7953	3.3309

**Table 4:** Adsorption energy densities of acids and the deformation energy.

	Formic	Acetic	Levulinic	Naphtenic	Stearic
*V* (cm^3^/mol)	39.42	57.10	113.40	264.85	350.76
*V* ([26])	37.912	57.577	103.573	--	313.898
*D* (kcal/cm^3^)	0.2909	0.2448	0.1714	0.0886	0.0762
∆E (kcal/mol)	6.995	8.7898	5.2644	−22.0311	−51.9484

**Table 5:** The solubility parameters of the acids ((J·cm^−3^)^0.5^).

	Cellulose	Formic	Acetic	Levulinic	Naphtenic	Stearic	Oil
*δ*	32	33.2134	28.6448	27.0623	19.7663	18.5987	16–19 *
*δ*_E_	--	23.4952	19.1791	15.9908	9.07736	7.81325	--
*δ*_V_	--	23.4702	21.2716	21.8312	17.5577	16.8772	--
*δ* ([26])	32	24.242	20.613	25.314	16.344	17.975	--

* Transformer mineral oil is a typical kind of alkane comprising 8~15 carbon atoms, and, according to reference [18], the solubility parameter of such a kind of alkane ranges from 16 to 19.

**Table 6:** The five acids detected in the transformer.

Name	Molecular Formula 1	Molecular Formula 2	Molecular Weight
Formic	HCOOH	CH_2_O_2_	46
Acetic	CH_3_COOH	C_2_H_4_O_2_	60
Levulinic	CH_3_COCH_2_CH_2_COOH	C_5_H_8_O_3_	116
Naphtenic	C_n_H_2n-1_COOH ^a^	C_n+1_H_2n_CO_2_	226
Stearic	CH_3_(CH_2_)_16_COOH	C_18_H_36_O_2_	284

^a^*n* = 13 in this paper.
